# Advanced Glycation End-Products and Their Effects on Gut Health

**DOI:** 10.3390/nu15020405

**Published:** 2023-01-13

**Authors:** Kate Phuong-Nguyen, Bryony A. McNeill, Kathryn Aston-Mourney, Leni R. Rivera

**Affiliations:** IMPACT, Institute for Innovation in Physical and Mental Health and Clinical Translation, Deakin University, Geelong 3220, Australia

**Keywords:** advanced glycation end-products, gastrointestinal tract, microbiome, enteric neurons, intestinal permeability, IBD

## Abstract

Dietary advanced glycation end-products (AGEs) are a heterogeneous group of compounds formed when reducing sugars are heated with proteins, amino acids, or lipids at high temperatures for a prolonged period. The presence and accumulation of AGEs in numerous cell types and tissues are known to be prevalent in the pathology of many diseases. Modern diets, which contain a high proportion of processed foods and therefore a high level of AGE, cause deleterious effects leading to a multitude of unregulated intracellular and extracellular signalling and inflammatory pathways. Currently, many studies focus on investigating the chemical and structural aspects of AGEs and how they affect the metabolism and the cardiovascular and renal systems. Studies have also shown that AGEs affect the digestive system. However, there is no complete picture of the implication of AGEs in this area. The gastrointestinal tract is not only the first and principal site for the digestion and absorption of dietary AGEs but also one of the most susceptible organs to AGEs, which may exert many local and systemic effects. In this review, we summarise the current evidence of the association between a high-AGE diet and poor health outcomes, with a special focus on the relationship between dietary AGEs and alterations in the gastrointestinal structure, modifications in enteric neurons, and microbiota reshaping.

## 1. Introduction

Advanced glycation end-products (AGEs) are a group of chemically heterogeneous compounds that are formed through the Maillard reaction when reducing sugars react non-enzymatically with amino groups in proteins, nucleic acids, and lipids [1]. These reactions are irreversible, resulting in the accumulation of AGEs in the blood and tissues over time. In humans, the accumulation of AGEs over the lifespan contributes to the normal age-related physiological decline and is directly involved in the pathogenesis of degenerative musculoskeletal conditions in older people, such as osteoporosis [2], osteoarthritis [3,4], and sarcopenia [5,6,7]. However, an increase in AGE accumulation related to modern dietary practices has been implicated in the dramatic rise in the prevalence of non-communicable diseases in younger people, such as diabetes [8,9,10], kidney disease [10,11,12], and mental health conditions [13,14,15,16]. AGEs in the body can be broadly divided into two groups, i.e., endogenous and exogenous, reflecting their origin. Endogenous AGEs are formed in the body as part of normal metabolic processes. As endogenous AGE production is directly related to blood glucose concentration, their production is accelerated in hyperglycaemic conditions such as diabetes and insulin resistance [17,18]. Exogenous AGEs are derived from external sources, primarily the diet, and are present in particularly high concentrations in foods which have been cooked at high temperatures, are highly processed, or have been prepared for long-term storage [19].

Although the negative health effects of AGEs have been well described in most body systems, their effects on the gastrointestinal tract have been largely overlooked. Within the gut, there is a strong body of evidence to indicate that AGEs negatively affect the gut and overall body health directly, through their effects on the gut architecture, as well as indirectly, through their interactions with the gut microbiota. This review will focus on the effects of AGEs on gastrointestinal structure, function, and microbiome, as well as identify knowledge gaps in the current literature.

## 2. Formation of Advanced Glycation End-Products (AGEs) 

**Figure 1:**
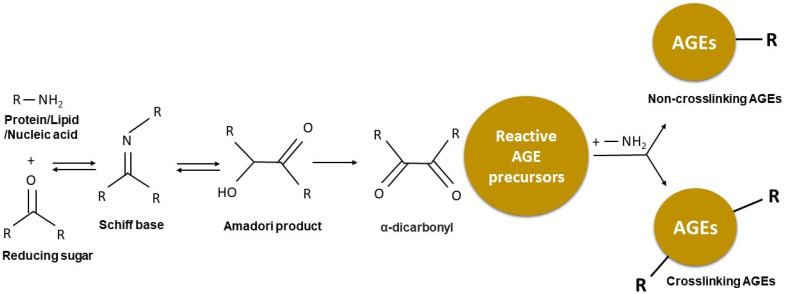
Overview of the Maillard reaction leading to the formation of advanced glycation end-products (AGEs). The Maillard reaction occurs when a carbonyl group is exposed to an amine, leading to a Schiff base formation, followed by Amadori rearrangement and formation of Amadori products. The Amadori products undergo irreversible oxidation, dehydration, enolisation, cyclisation, and fragmentation that lead to the formation of reactive intermediate AGE precursors. Reactive AGE precursors interact with protein-bound lysine or arginine to form AGEs, which can be classified as crosslinking or non-crosslinking.

The Maillard reaction is a complex reaction that occurs when the free amino groups of proteins, lipids, or nucleic acids are heated in the presence of reducing sugars (glucose, lactose, fructose, and maltose). This condensation reaction leads to the formation of an unstable Schiff base (early glycation product), which spontaneously undergoes a process of intramolecular degradation, known as the Amadori rearrangement, resulting in the formation of Amadori products (intermediate glycation products) [20,21,22]. The chemical arrangement of the Amadori products is more stable than that of Schiff bases, although this reaction is reversible as these products are highly unsaturated, making them susceptible to polymerisation [23,24]. In the final stage, the Amadori products undergo irreversible chemical rearrangements including oxidation, dehydration, enolisation, cyclisation, and fragmentation to produce diverse classes of reactive intermediates including reactive AGE precursors [3,25]. These reactive AGE precursors can interact with protein-bound lysine or arginine, leading to the formation of AGEs (Figure 1).

While the majority of AGEs are produced by the non-enzymatic Maillard reaction [26,27], some AGEs are formed via alternative pathways such as autoxidation of glucose and peroxidation of lipids [28,29,30,31,32], or via the polyol pathway. In this pathway, glucose is converted first to sorbitol by the enzyme aldose reductase and then to fructose by the action of sorbitol dehydrogenase. After that, metabolites of fructose (such as fructose-3-phosphate) are converted into α-oxaldehydes, which give rise to several reactive dicarbonyl derivatives (reactive AGE precursors), including glyoxal, methylglyoxal, and 3-deoxyglucasone [33], which bind to intracellular and extracellular proteins and DNA to modify them, producing AGEs. 

## 3. Sources of AGEs

### 3.1. Endogenous AGEs

Endogenous AGEs are formed intracellularly and extracellularly in all tissues and body fluids, when the normal sugar metabolism in the cells and circulating glucose form covalent adducts with plasma proteins through the process of glycation, which undergo multiple steps of the Maillard reactions described in the previous section, to produce AGEs [26,34]. The rate of endogenous AGE production is determined by genetics [35,36], age [37,38], and circulating glucose concentration [39]. 

### 3.2. Dietary-Derived (Exogenous) AGEs

There has been increasing evidence supporting the significant contribution of AGEs derived from exogenous sources, primarily diets, to the accumulation of AGEs in tissues and their circulation throughout the body. These exogenous AGEs are hence recognised as a major contributing source to the total body pool of AGEs [40,41,42]. The AGE content of the diet can vary significantly, depending on the types of foods consumed and their preparation methods. Food of animal origin and those which have been exposed to high heat or alkaline conditions during preparation are particularly high in AGEs [29,40]. High processing temperatures are required for ensuring food safety, as well as to increase the flavour and appearance of food. Consequently, dietary AGE consumption is particularly high in the modern Western diet [43,44,45]. In contrast, food prepared using cooking methods which use lower heat, shorter cooking time, and higher humidity (e.g., boiling, steaming) are associated with much lower AGE concentrations [19,40,46,47,48].

In addition to dietary AGE consumption, exposure to tobacco products can be a significant source of exogenous AGEs. Cured tobacco and tobacco smoke contain highly reactive glycation products leading to the formation of AGEs [49], which is reflected by higher circulating concentrations of AGEs in smokers compared with non-smokers [50,51,52].

In summary, the AGE concentration in the blood and tissue is dependent on dietary intake, genetics, age, and circulating glucose concentration. Furthermore, there is growing evidence that AGEs derived from multiple sources may act synergistically to contribute to a range of negative health outcomes [43]. A high total body AGE content, regardless of whether it is endogenous or dietary in origin, is associated with a wide range of negative health outcomes and with increased chronic disease burden affecting numerous sites in the body, including the gastrointestinal system.

## 4. AGEs, Oxidative Stress, and Inflammation in the Gut

Studies have shown that the accumulation of AGEs within the gut promotes an influx of macrophages [53,54] triggering a local inflammatory response characterised by high concentrations of pro-inflammatory cytokines and reactive oxygen species. Inflammatory bowel disease (IBD), an umbrella term which encompasses both ulcerative colitis and Crohn’s disease, is a potentially debilitating condition that presents with a range of gastrointestinal symptoms including diarrhoea, abdominal pain, and rectal bleeding [55]. Although the causes of IBD are multifactorial [56], there are currently limited studies looking at the direct effects of AGEs in IBD. However, there is evidence indicating the effects of AGEs in the worsening pathogenesis of related diseases (such as diabetes), particularly in increased inflammation in the gut, implying a mounting body of evidence that AGEs are a contributing factor to the development of IBD [57,58,59,60].

### 4.1. The Role of AGEs in the Pathophysiology of IBD

There is an established relationship between AGE and its receptor, the receptor for advanced glycation end-products (RAGE), signalling and IBD, primarily in the form of correlational data. For example, a study by Kato et al. [61] indicated a direct correlation between the concentration of the AGE pentosidine and that of u8-OHdG, a biomarker of oxidative DNA damage and tissue damage, in samples of inflamed gastrointestinal tissue from human subjects with IBD [61]. Furthermore, biopsy samples from people with IBD showed that RAGE-mediated NF-kB activation was higher in areas of inflamed tissue than in areas of non-inflamed gut tissue [62,63]. Of note, the accuracy of the ELISA method used by Kato et al. [61] and Andrassy et al. [62] has been questioned for the reliability of AGE measurement, considering the potential detection of additional contaminants [64,65]. Moreover, the association between RAGEs and ulcerative colitis reported by Andrassy et al. [62] is based on findings from a very small sample, indicating that further research in this area is warranted. Recently, a large prospective cohort study reported that the risk of developing IBD was positively correlated with the intake of ultra-processed foods [66,67]. Although this study did not specifically assess the AGE content of the diet, consumption of soft drinks, refined sweetened foods, and processed meats was associated with an increased hazard ratio for the development of IBD [66]. Given that these food groups either have a high AGE content (processed meats [19]) or promote the formation of AGEs through the endogenous pathway (soft drinks [68]), the total AGE (endogenous and exogenous) intake is considered higher with the ultra-processed diet [19,69]. Collectively, the data from these studies indicate that there is a potential relationship between AGE/RAGE accumulation in the gastrointestinal tract and the inflammatory symptoms of IBD. 

Alongside the available human data, studies using relevant animal models have provided experimental support for an association between AGEs and IBD and provide an important insight into the mechanisms by which AGEs act to promote inflammation and oxidative stress in the gut. Importantly, animal models provide an opportunity to study the changes which occur in the gut prior to the onset of clinical disease. In this regard, Bramhall et al. [70] found that upregulated RAGE expression is a marker of colitis susceptibility, using the AKR mouse model of IBD. Another mouse model, the RAGE-knockout mouse, is protected from developing enterocolitis and colitis in the presence of the AGEs indomethacin, dextran sulphate sodium (DSS), and trinitrobenzene sulfonic acid (TNBS) [71]. Additionally, it is well established that upregulated NF-κB signalling is associated with the development of IBD [72,73,74,75] and gastrointestinal tumorigenesis [76]. Remarkably, a study by Nass et al. [77] indicated that transgenic mice expressing the firefly reporter gene under the control of an NF-κB-responsive promoter showed luciferase activity in the gut after injection of AGE-BSA. This implies a significant induction of NF-κB signalling in the gut after the ingestion of dietary AGEs proposedly due to an elevated AGE/RAGE interaction [77]. However, it should also be noted that AGE is only one of many RAGE pro-inflammatory ligands (such as S100 proteins and HMGB1), some of which bind to RAGE with stronger affinities in comparison to AGEs [78]. Particularly, increased interaction between RAGE and S100 proteins (S100A, S100A9, and S100A12) is associated with increased inflammation [79,80], contributing to worsen the pathogenesis of IBD [81,82,83,84,85,86]. Similarly, HMGB1 is another ligand binding to RAGE [80] and known to be a biomarker of IBD in numerous animal studies [87,88,89] and human studies [90,91]. There is also evidence that AGEs increase the expression of S100 proteins [92] and the activation of HMGB1 signals [93] to induce increased inflammation. These findings lend further support to the potential role of a direct AGE/RAGE interaction and an indirect interaction of AGEs and other RAGE ligands contributing to the pathogenesis of IBD. However, more studies (such as on S100 and HMGB1 knockout models) are required to have a better understanding of the important involvement of AGEs in the development of IBD.

The manipulation of dietary AGE intake and the examination of the associated effects on gastrointestinal structure and function have also been valuable for examining the relationship between AGE intake and IBD. Overall, the findings of these studies indicate that a high dietary AGE intake is associated with an increase in oxidative stress and the development of a pro-inflammatory environment consistent with IBD. For example, macrophage infiltration, inflammation, and increased oxidative stress within the colon have been reported in several studies in which rats and mice were fed a high-AGE diet [53,54,94]. Additionally, an in vitro study by van der Lugt et al. exposing human macrophage-like cells to dietary AGEs led to a significant induction of TNF-alpha secretion, which was later shown to be reduced by the addition of a RAGE antagonist (carboxymethyllysine antibodies), further supporting a relationship between high AGE intake and inflammation [64]. Moreover, there have been some reports suggesting that a high AGEs intake might directly worsen inflammation. For example, studies indicated that high consumption of dietary AGEs from an extensively hydrolysed formula by infants resulted in not only an elevated circulation of the body’s AGE pool [95] but also a significant increase in intestinal permeability compared to the consumption of a conventional formula or breast milk [96]. To date, the current literature has questioned many findings from animal and human studies due to the unreliability of the methods used for AGEs measurements. Most studies in the past predominantly measured AGE levels in foods using ELISA [61,62,97,98,99,100,101,102,103] instead of ultraperformance liquid chromatography–tandem mass spectrometry (UPLC-MS/MS), which is currently considered the gold standard for AGE measurements [19,104]. In addition, many studies focused on producing high-AGE and low-AGE diets using high and low baking (heat-treated) methods or changing the cooking technique (boiling, poaching instead of grilling, frying) to adjust the AGE concentrations in foods. It is worth noting that changing the cooking method to alter the AGE content may lead to alterations in nutrients and caloric intake, which can potentially contribute to the effects of AGEs in IBD [99,100,101,103,105,106,107,108]

### 4.2. Dietary Approaches to Reducing AGE-Related Inflammation in the Gut

Given that AGE/RAGE signalling contributes to the onset and severity of inflammatory conditions of the gut, attenuating triggers of RAGE activation and/or lowering AGE accumulation have been proposed as potential treatments for IBD [71]. While there are not many reports directly investigating the immediate effects of AGEs consumption in the context of IBD, the effect of dietary AGEs in diabetes has been widely investigated [109,110]. There has been increasing evidence indicating that diabetes drives the endogenous formation of AGEs and promotes intestinal barrier disturbance and dysfunction [54,110,111,112]. As studies indicate the beneficial effects of low-AGE diets in improving diabetic complications, this might be proposed as a strategic treatment to improve IBD. For example, a human study by Luévano-Contreras et al. [103] suggested that human subjects who consumed a low-AGE diet instead of the standard diet (recommended by the American Diabetes Association) for 6 weeks had significantly lower circulating concentrations of inflammatory markers and oxidative stress. Moreover, a meta-analysis conducted by Baye et al. [113] also indicated the positive effects of low-AGE diets in reducing inflammation and oxidative stress, regardless of the diabetes status. Collectively, the data from these studies suggest diets low in AGE content might be a viable strategy for reducing inflammation in the context of IBD.

Although the majority of studies indicate pro-inflammatory effects of AGEs in the gut, it is possible that specific AGEs can be anti-inflammatory, but further research is required. For example, a human study by Dittrich et al. [114] reported that the consumption of certain high-AGE-containing foods (bread crust, dark beer, and coffee) is associated with the protective effects of increased oxidative resistance of human low-density lipoprotein in plasma. These findings highlight the potential diverse effects of AGEs and other Maillard reaction products. However, in the context of IBD, the available evidence suggests potential detrimental effects of AGEs. Nevertheless, more studies with validated methodologies (such as UPLC–MS/MS) are needed to verify the existing knowledge base of AGE measurements (which were previously detected using ELISA) and AGE implications on gut health.

## 5. AGEs and the Gut Barrier

The intestinal barrier is specialised to maintain homeostasis in the internal environment and to fulfil two seemingly opposing roles: (1) to maintain a peaceful co-existence with numerous intestinal symbionts without provoking chronic inflammation and (2) to provide measured inflammatory and defence mechanisms as part of the immune response [115]. The intestinal barrier is composed of a single layer of enterocytes with several intermolecular protein structures between each cell, including tight junction proteins. Tight junction proteins (e.g., claudins, occludins, tricellulin, and zona occludens family) are found towards the apical end of the enterocyte and function as a seal between neighbouring enterocytes to maintain the barrier integrity. Adherens junction proteins, in particular, E-cadherin, also play an important role in maintaining barrier integrity [116,117]. 

To date, there has been increasing evidence suggesting a high AGEs intake might be causative to elevated intestinal disruption, increased intestinal permeability, and high levels of inflammatory markers in the gut. AGEs and RAGE are highly expressed in the epithelial cells of the gastrointestinal tract [57,58,59]. In streptozotocin-induced diabetic rats, the detection of AGEs and RAGE significantly increased in the small and large intestines [57,58]. Furthermore, previous studies of diabetic rats showed that brush border membrane fluidity is decreased by oxidative damage and is correlated with an increase in AGE in the duodenum (28%), jejunum (94%), and ileum (58%) [118]. The increased expression of RAGE might enhance the ligation of AGEs to RAGE, consequently accelerating the localised production of reactive oxygen species (ROS). Increased expression of RAGE and ROS is known to be closely involved in disrupting the integrity of intercellular tight junctions, which can compromise the integrity of the gut barrier, leading to increased gut permeability [119]. ROS production may also increase inflammation in intestinal epithelial cells and contribute to increased gut permeability [120].

Elevated levels of AGEs have been directly implicated in increased gut permeability both in vitro and in vivo. Using an in vitro model of the human intestinal epithelium, Guibourdenche et al. [121] demonstrated reduced tight junction and mucin gene expression in the intestinal mucosa following a 6 h exposure to AGEs (carboxymethyllysine and acrylamide), with no significant changes in paracellular intestinal permeability measured by changes in FITC-Dextran efflux. In other studies by Shi et al. [122,123], the treatment of small intestinal epithelial cells (IEC-6 cells) with glycated caseinate hydrolysates led to a reduced expression of tight junction proteins and increased intestinal permeability, measured by changes in transepithelial resistance. The discrepancy in the changes in intestinal permeability may be due to the different methods of measurement (FITC-dextran efflux versus transepithelial electrical resistance), different cell models, different treatment times, as well as various AGE concentrations.

Recently, Snelson et al. [54] showed that rats fed a high AGE diet for 24 weeks had altered expression of tight junction proteins in the jejunum (reduced occludin and claudin-1; increased claudin-5), ileum (increased claudin-1 and claudin-5; reduced occludin), and colon (increased claudin-1 and claudin-5; reduced occludin). Additionally, a study by Qu et al. [124] investigated the changes in colonic permeability in rats fed a high-AGE diet. A histological examination of the colon revealed significant alterations in the colonic structure of rats fed a high AGE diet for 18 weeks, including crypt loss and distortion, reduced goblet cells, increased dysplasia, and mucosal thickening with no obvious inflammatory infiltration. The expression levels of the tight junction proteins occludin and zonula occludens-1 were also significantly decreased, suggesting increased colonic permeability [124]. Of note, in both humans and rats, an increase in serum LPS was reported following the consumption of a high-AGE diet. Specifically, Pendya et al. [125] indicated a 71% increase in plasma lipopolysaccharide (LPS) in human subjects consuming a high-AGE diet after only 4 weeks, and the previously mentioned rat study by Snelson et al. [54] also noted an increase in serum LPS after 24 weeks on a high-AGE diet. In this study, the increase in LPS was reversed by treatment with alagebrium (an AGE inhibitor) and the C5aR1 inhibitor (complement signalling inhibitor), indicating that AGE signalling may mediate this increase in LPS. However, the levels of plasma LPS in the animal study by Qu et al. [124] were modestly but not significantly increased in rats after 18 weeks of consuming a high-AGE diet. LPS are bacterial glycolipids found on the surface of most Gram-negative bacteria in the gut [126,127] and are known to be macrophage activators triggering cellular signals for chronic inflammation [128,129]. It is established that an increased level of LPS is correlated with increased intestinal permeability and epithelial damage [127]. Overall, these findings highlight the pro-inflammatory effects of AGEs in the gut and their pathogenic actions, altering the gut barrier structure and promoting gut leakiness. 

## 6. AGEs and Enteric Neurons

The enteric nervous system (ENS) is composed of nerve plexuses, which include neurons, axons, and enteric glial cells [130]. In the intestine, most neurons are found in two ganglionated plexuses, the myenteric plexus, which is mainly involved in coordinating motility, and the submucosal plexus, which is largely involved in the control of mucosal function and blood flow [131]. There are approximately twenty different types of neurons including motor neurons, interneurons, and intrinsic primary afferent neurons (also referred to as intrinsic sensory neurons) [131]. These enteric neurons substantially form circuits capable of autonomic reflex activity to predominantly direct the functions of the gut [132,133]. Therefore, any loss or damage to enteric neurons may result in gastrointestinal dysfunction [134,135] and contribute to the pathology of many gastrointestinal diseases [136]. To date, there has been increasing evidence suggesting that an excessive AGEs intake might lead to the loss of enteric neurons and an alteration in nitrergic signalling in the ENS.

RAGE has been shown to be expressed in myenteric [58,137] and submucosal [58] neurons in the oesophagus and intestine. The expression of RAGE in the ENS is also enhanced in diabetic conditions, suggesting the involvement of the AGE/RAGE interaction in gastrointestinal-related diabetic neuropathy [58]. 

There is also good evidence that AGEs alter the nitrergic signalling in the ENS. Nitric oxide (NO) is a free radical involved in numerous biological functions, including vasodilation [138], transmission from inhibitory neurons to the gut muscle, modulation of neurotransmission, inhibition of platelet aggregation, and inhibition of smooth muscle proliferation [138,139]. NO is synthesised from L-arginine by the activity of nitric oxide synthases (NOS) [138,140,141]. All three isoforms of NOS have been identified in the intestine: neuronal NOS (nNOS/NOS1), endothelial NOS (eNOS/NOS3), and inducible NOS (iNOS/NOS2) [141]. NO produced by nNOS is a transmitter of the inhibitory neurons supplying the muscle of the gastrointestinal tract. A rat study by Korenaga et al. [137] indicated that AGEs suppress the expression of nNOS in vitro via RAGE. In a follow-up study, the authors showed a significant reduction in nNOS expression in the myenteric plexus of the duodenum of diabetic rats, which was reversed by inhibiting the formation of AGE using aminoguanidine (a compound established to block the formation of AGEs) and ALT-711 (a compound breaking the cross-links of AGEs) [137,142]. In line with this finding, Voukali et al. [143] demonstrated that exposure to high levels of AGEs only resulted in decreased nNOS-positive neurons in the myenteric plexus, with no effect on vasointestinal peptide- or calbindin-positive neurons. 

The literature regarding the mechanisms of action of the AGE/RAGE interaction in the ENS is limited, despite the central role that the ENS plays in coordinating and regulating gut function. Furthermore, most studies have only focused on changes in nNOS expression in the myenteric plexus, while very little is known about what happens to other subtypes of enteric neurons in the myenteric and submucosal plexuses which are affected by AGEs. As discussed above, there is a correlation between high levels of AGEs and increased gut permeability. Therefore, it is possible that damage to the enteric neurons is linked to increased gut permeability, given that the enteric neurons are involved in the regulation of the intestinal barrier function [144]. 

## 7. AGEs and the Gut Microbiota

The gastrointestinal tract is host to trillions of microorganisms, collectively known as the gut microbiota. A substantial body of evidence supports the pivotal role that the gut microbiota plays in maintaining host health. A large proportion of dietary AGEs cannot be absorbed in the small intestine, and as a result, these compounds pass through to the large intestine where they may be partially degraded and metabolised by the gut microbiota [145]. It is well established that excessive AGE consumption can lead to deleterious health outcomes; therefore, it is important to understand how AGEs affect the gut microbiota composition, given that these compounds are abundantly present in the modern diet. 

### 7.1. Human Studies

There are limited studies in humans investigating the effects of AGEs on the gut microbiome composition. One of the early human studies conducted by Seiquer et al. [146] randomised the trialing effects of a high-AGE diet on the gut microbiota composition in adolescents for 2 weeks. Their study indicated that adolescents consuming a high-AGE diet had a significantly lower level of Lactobacilli. The reduction in the relative abundance of Lactobacilli is not likely to be beneficial, because this is a Gram-positive lactic acid bacterium [147] known to play key roles in protecting the intestinal barrier [148] and in maintaining microbiota homeostasis [149,150]. Yacoub et al. [101] conducted a randomised open-label-controlled trial, looking at the association between the gut microbiota and the consumption of dietary AGEs in patients with end-stage renal disease. Their study indicated that a one-month dietary AGE restriction was associated with a decline in the relative abundance of *Prevotella copri* and *Bifidobacterium animalis* and with an increase in the relative abundance of *Alistipes indistinctus, Clostridium citroniae, Clostridium hathewayi*, and *Ruminociccus gauvreauii* [101,102,151,152]. The increase in *Alistipes indistinctus* is not likely beneficial, as it is recognised to be highly pathogenic due to its strong association with colitis and tumorigenesis in mice [153]. Further, the decrease in *Prevotella copri* may also be detrimental, as this is known to generate high levels of short-chain fatty acids (SCFAs) [102,124,149,150,154,155,156]. SCFAs are recognised to exert multiple health benefits, in particular improved intestinal barrier function [155,156,157]. Recently, a randomised controlled trial conducted by Linkens et al. [158] with a large number of participants who were abdominally obese but otherwise healthy investigated the effect of low and high AGEs intakes for 4 weeks on the gut microbiota composition. While the researchers indicated no differences in microbial diversity, richness, and overall microbiota composition between the two dietary treatments, their PERMANOVA analysis revealed that a 4-week dietary AGE restriction significantly reduced the relative abundance of Anaerostipes and Oscillibacte and increased the relative abundance of Tyzzerella, Family_XIII_UCG-001. The reduction in the relative abundance of Anaerostipes and Oscillibacter is likely to be unfavourable because Anaerostipes is a SCFA-producing genus [159] and Oscillibacter is associated with increased insulin sensitivity [160]. In contrast, the enrichment of Tyzzerella and Family_XIII_UCG-001 is identified to be associated with chronic intestinal inflammation and an increased risk of irritable bowel syndrome [161] and IBD development [162,163]. Together, the gut microbiota dysbiosis observed in these studies might suggest potential roles of dietary AGEs in markedly reshaping the microbiota profile; however, consuming a low-AGE diet in such a short period might not show protective effects in improving the gut microbiome. Moreover, these results also indicate that multiple environmental conditions and/or co-morbidities could have taken place beyond the dietary AGE consumption to reduce gut microbiota dysbiosis and improve gut health. 

### 7.2. Animal Studies

There are numerous animal studies that investigated diets that are high in AGEs and their effects on the gut microbiota composition. Although there are conflicting findings, many studies found reduced gut microbiota diversity and richness [102,151,152], a reduced level of butyrate-producing bacteria, [102,151,157,164], and increased levels of Desulfobrivio [102,124,154], Lachnospiraceae [157] and Dubosiella [165] following exposure to high-AGE diets. 

To investigate the relationship between AGE intake and the gut microbiota, Wang et al. [102] exposed mice to two high-AGE diets, i.e., a heat-treated diet and a diet enriched with exogenous AGEs for 24 weeks, and assessed the microbiome composition. This study found a reduced abundance of Bacteroidales_S24-7, Bacteroidaceae, Porphyromonadaceae, Odoribacteraceae, Lachnospiraceae, Rikenellaceae, and Erysipelotrichaceae and an increased abundance of Desulfovibrionaceae in mice which were fed high-AGE diets. They also looked at co-abundance groups (CAGs), which aggregate individual microbiome members into functional ecological units. Microbiome members were grouped together if they utilised similar resources or worked together as a functional group. The authors found that CAG1/2/3/4/5 was decreased in mice fed exogenous and dietary AGEs. Interestingly, these CAGs contained Operational Taxonomic Units from Bacteroidales_S24-7, Ruminococcaceae, and Lachnospiraceae, which are butyrate-producing bacteria. Butyrate is the major energy source of enterocytes, and a reduction in butyrate-producing bacteria can be associated with impaired epithelial barrier function and increased inflammatory and oxidative stress [102]. In addition, Qu et al. [124] showed that high-AGE diets in rats resulted in a dramatic loss of beneficial bacteria, including Ruminococcaceae, Lachnospiraceae, Alloprevotella, and Butyrivibrio. Further, an increased AGE content led to enhanced protein fermentation, as evidenced by the elevated concentration of branched-chain fatty acids (BCFAs) and ammonia in the colon. Colonic protein fermentation is deemed to be detrimental to host health due to toxic and harmful products such as phenolic, sulphur, indoxyl sulphate, and ammonia, implicating adverse effects on gut health by diminishing the energy supply to colonocytes [124]. Together, these findings highlight the pivotal role of AGEs in markedly altering the gut microbiota homeostasis and in promoting the microbiota dysbiosis.

## 8. Conclusions

**Figure 2:**
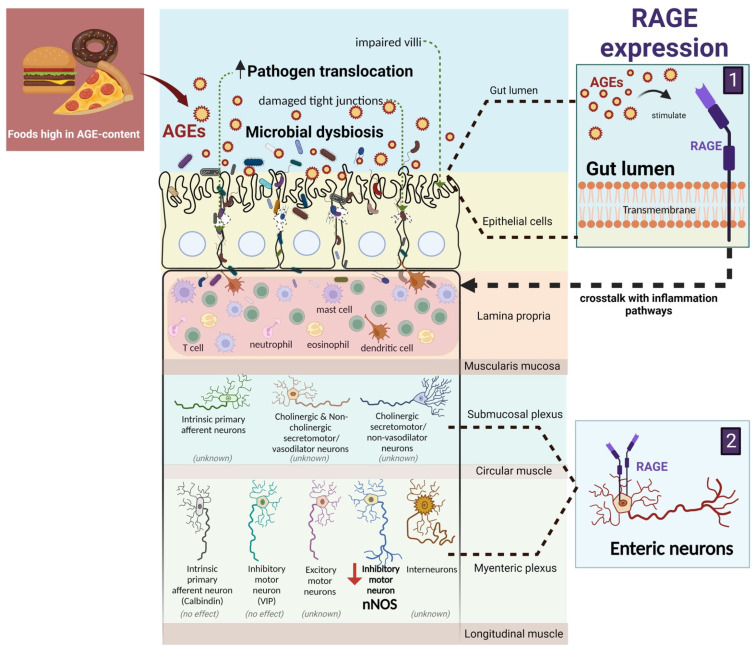
Overview of the effects of dietary AGEs on the gut. AGEs are ligands for various cell surface receptors, of which the receptor for AGEs (RAGE) is the main one and has been extensively studied. RAGE is expressed in the intestinal epithelium and the enteric neurons of the submucosal plexus and myenteric plexus. There has been growing interest indicating the potential roles of endogenous and exogenous AGEs that may act synergistically to accelerate the pathogenetic effects associated with AGEs. AN excessive dietary AGE intake is associated with alterations in the gut structure leading to enhanced gut barrier dysfunction, changes in the expression of enteric neurons, microbial dysbiosis, and inflammation (Created with BioRender.com).

Dietary AGEs are highly prevalent in the modern diet, with increased total endogenous and exogenous AGE formation contributing to the pathogenesis of many diseases. In this review, we provided evidence of the deleterious effects of AGEs on the gastrointestinal tract, specifically, their contribution to markedly altering the gut structure leading to increased intestinal permeability and reduced expression of enteric neurons, as well as to reshaping the microbiota composition (Figure 2). The crosstalk between AGEs and the gut signifies important influences in local and systemic effects on overall human health. However, little is known about the molecular mechanisms in the gut involved in absorbing the dietary AGEs and the potential intestinal niches influencing the gut structure and function. Additionally, reducing the AGE intake may be beneficial to improve gut health, given how prevalent AGEs are in the modern diet. Thus, more human studies with validated analytical methods (such as UPCL–MS/MS or a better gold standard for AGE measurements in the future) are needed to support the existing knowledge base of high AGE exposure to the gut, which is primarily derived from animal studies, and to determine the long-term effects of a low AGE consumption in people experiencing gastrointestinal disorders and their co-morbidities.

## Data Availability

No new data were created or analyzed in this study. Data sharing is not applicable to this article.
